# Buffalo Academy of Medicine

**Published:** 1903-11

**Authors:** Wm. Irving Thornton


### SOCIETY PROCEEDINGS.

### Buffalo Academy of Medicine

*Section on Medicine.*

REPORTED BY WM. IRVING THORNTON, M. D., Secretary.

THE regular meeting of the Section of Medicine of the Buffalo Academy of Medicine, was held in the Academy rooms, Public Library Building, Tuesday, September 15, 1903. The meeting was called to order at 8.45 p. m. by the chairman, DR. ARTHUR W. HURD.

DR. GEO. E. FELL presented a paper entitled,

LAKE AND RIVER CURRENT OBSERVATIONS AND THEIR VALUE TO OUR CITY.

The occasion for this investigation was the epidemic of typhoid fever at the Steel Plant district, which is drained by Smokes Creek, whose waters, it was feared, polluted Buffalo's water supply; also for the reason that a new intake pier at the Horseshoe Reef was being contemplated.

A number of floats were placed in the lake, one a mile out from Smokes Creek; others at various positions outside the breakwater, one of the latter being one thousand feet west of the red light. The first passed between the breakwalls into the inner harbor. The latter floated down the river, 320 feet east of the intake pier; another float started at a point at the intersection of the projections of Jersey Street and the old breakwall and passed into the intake pier. This point, however, in the judgment of the essayist, was too far out to be contaminated by Smokes Creek. He, therefore, concluded that Buffalo's water supply was not endangered because of typhoid at the Steel Plant.

DR. A. L. BENEDICT read a paper entitled,

THE HAMBURG FILTER PLANT AND FILTRATION OF WATER IN HOLLAND, giving an outline of the construction of the filter plant and method of cleansing the same.

In discussing Dr. Fell's paper, DR HOPKINS said we should consider that Lake Erie is very superficial, and that there are many special currents differing from the general currents, as observed by the essayist, which might cause the water from Smokes Creek to contaminate our city water. While he believed that part of the water passed between the breakwalls into the inner harbor, he did not consider it possible for the entire volume to do so, as stated by Dr. Fell; and, therefore, did not believe the observations were conclusive.

Dr. W. D. Greene stated that the colon bacillus, which indicates contamination, had not been found in Buffalo's water supply for ten years previous to 1903, and that last year there was 45 per cent. more typhoid than usual. He stated that the water supply at the present time was good.

Dr. Bissell sent word that he was unable to be present and discuss Dr. Fell's paper.

Dr. Benedict believed that the reason we did not have a severe typhoid epidemic in the spring of this year was because we did not have the extensive freshets to wash the sewage from Smokes Creek far out into the lake, and also because there was a sand bar across the mouth of the creek.

Dr. Eugene Wasdin stated that there was an irresistible hydrostatic pressure forcing the superficial waters of Lake Erie through the openings in the breakwalls towards Buffalo's shore. He believed Dr. Fell proved his case.

Dr. A. W. Hurd suggested that the banking up of ice at the breakwall, during the winter, might deflect the general currents.

Dr. P. W. Van Peyma stated that a greater number of floats should have been used. He did not believe the observations were conclusive.

Dr. Jewett asked for an explanation of the discoloration, occasionally seen far out in the river.

Dr. Fell stated that this was due to a disturbance of the silt at the bottom of the lake and river, due to a storm.

Dr. E. E. Blaauw made some remarks relative to the Hamburg filter plant.

The meeting adjourned at 10.30 p. m.

Fellows present, 24.

---

*Stated meeting held October 6, 1903.*

Reported by E. A. BOWERMAN, M. D., Secretary.

The meeting was called to order at 8.45 p.m., by the president, Dr. Joseph W. Grosvenor.

The minutes of the last stated meeting held May 26, 1903, and special meeting held September 8, 1903, were read and approved.

The council recommended to the Academy the election of the following: Drs. E. J. Kiepe, Joseph Burke, Joseph Spangenthal,

William J. O'Donnell, Robert E. De Ceu, James A. McLeod, John Chalmers, James H. Carr, Nelson G. Russell, P. H. Hourigan and Richard J. Gould.

On motion, the secretary was instructed to cast a ballot for their election. They were declared elected.

Dr. M. Hartwig presented a case of a large cystic tumor over the left eyebrow, from which mucoidal material could be pressed into the nose. This was thought to communicate with the ethmoidal or frontal sinuses.

Dr. Marshall Clinton presented a case of acute intestinal obstruction, due to an inflamed Meckel's diverticulum in which a portion of the intestine had been resected.

Dr. Eugene A. Smith read a paper in which he reported three cases of cerebral compression, due to traumatic extradural hemorrhage, resulting from fracture of the skull, in which surgical intervention had resulted favorably.

Dr. M. Hartwig presented a similar case in which there had been both extra- and intradural hemorrhage, with recovery after operation. These cases were discussed by Dr. James W. Putnam.

Dr. Roswell Park reported two anomalous cases of gangrene of the entire large and small intestine. In each case the trouble developed suddenly without apparent cause, the patients being, before the onset of symptoms, apparently healthy and about their usual vocations. The symptoms were intense abdominal pain of a few hours duration, followed by vomiting, colapse and abdominal distention, the pathology being either an embolism of the mesenteric arteries or thrombosis of the mesenteric veins. One patient lived about 24 hours after the onset of symptoms; the other about 5 days. These cases were discussed by Drs. Blaauw, Benedict, Howell and Rooth.

Dr. Charles S. Jewett, the chairman of the membership committee, urged the coöperation of the members in increasing the membership of the Academy.

Adjourned at 10.15 p.m. Attendance, 54.

